# Morphological and viscoelastic properties of the Achilles tendon in the forefoot, rearfoot strike runners, and non-runners *in vivo*

**DOI:** 10.3389/fphys.2023.1256908

**Published:** 2023-09-07

**Authors:** Xini Zhang, Liqin Deng, Songlin Xiao, Weijie Fu

**Affiliations:** ^1^ Faculty of Sports Science, Ningbo University, Ningbo, China; ^2^ Research Academy of Grand Health, Ningbo University, Ningbo, China; ^3^ Key Laboratory of Exercise and Health Sciences of Ministry of Education, Shanghai University of Sport, Shanghai, China

**Keywords:** Achilles tendon, foot strike patterns, cross-sectional area, hysteresis, running

## Abstract

The purpose of this study was to investigate the differences in the morphological and viscoelastic properties of the Achilles tendon (AT) among different groups (rearfoot strikers vs. forefoot strikers vs. non-runners). Thirty healthy men were recruited, including habitual forefoot strike runners (*n* = 10), rearfoot strike runners (*n* = 10), and individuals with no running habits (*n* = 10). The AT morphological properties (cross-sectional area and length) were captured by using an ultrasound device. The real-time ultrasound video of displacement changes at the medial head of the gastrocnemius and the AT junction during maximal voluntary isometric contraction and the plantar flexion moment of the ankle was obtained simultaneously by connecting the ultrasound device and isokinetic dynamometer via an external synchronisation box. The results indicated that male runners who habitually forefoot strike exhibited significantly lower AT hysteresis than male non-runners (*p* < 0.05). Furthermore, a greater peak AT force during maximal voluntary contraction was observed in forefoot strike male runners compared to that in male individuals with no running habits (*p* < 0.05). However, foot strike patterns were not related to AT properties in recreational male runners (*p* > 0.05). The lower AT hysteresis in male FFS runners implied that long-term forefoot strike patterns could enhance male-specific AT’s ability to store and release elastic energy efficiently during running, resulting in a more effective stretch-shortening cycle. The greater peak AT force in male FFS runners indicated a stronger Achilles tendon.

## 1 Introduction

To adapt to bipedal running, humans have gradually evolved the Achilles tendon (AT), which serves as a crucial structure connecting the heel and the plantar flexor muscles of the foot. It plays a primary role in transmitting the muscular force generated by the calf muscles during the movement, enabling efficient force or energy storage (during contact) and release (during push-off) in the lower limb during running and jumping (Bramble and Lieberman, 2004; Lorimer and Hume, 2016). Achilles tendinopathy accounted for the highest proportion of injury incidence (10.3%) in runners (Davis et al., 2017), with a cumulative lifetime prevalence of 52% in endurance runners (Lorimer and Hume, 2016). Particularly, the running-related injury incidence proportion of Achilles tendinopathy is male-biased (Francis et al., 2019), and the majority of AT ruptures occurred in male compared with female patients, with an incidence rate ratio ranging from 5.5:1 to 30:1 (Möller et al., 1996; Longo et al., 2009; Lemme et al., 2018). Thus, understanding the biomechanical properties of the AT and its response to different running habits, particularly in the male-specific AT, is important for optimizing training strategies and minimizing the risk of injuries (Chen et al., 2023).

Running is a popular form of exercise that can significantly impact the musculoskeletal system, including AT. Different foot strike patterns, such as rearfoot strike (RFS) and forefoot strike (FFS), have been observed in runners. Rearfoot strikers primarily contact the ground on their heels, whereas forefoot strikers initially contact the ground with the ball of their foot before the heel comes down (Hayes and Caplan, 2012). The biomechanical implications of different foot strike patterns on the AT have been a topic of interest in the field of sports science, and most studies have investigated the effect of foot strike patterns on AT’s morphological and mechanical properties as a primary outcome. However, previous studies have yielded controversial results. Some studies have found that FFS runners had a greater cross-sectional area (CSA) of the AT (Histen et al., 2017; Hirono et al., 2022), whereas others have not observed this significant difference (Kubo et al., 2015; Kernozek et al., 2018). Furthermore, whether the high AT load in FFS runners during running indicates improved running performance or increased risk of Achilles tendinopathy is currently a subject of debate (Rice and Patel, 2017; Kernozek et al., 2018).

Tendons are primarily composed of a collagenous matrix with elastic properties, which enable them to efficiently transmit force according to the amount of stretch they experience (Peltonen et al., 2013). However, viscoelasticity, an important characteristic, has often been overlooked in previous assessments comparing the AT mechanical properties of runners with different foot strike patterns. Currently, only a limited number of studies have investigated the *in vivo* viscoelasticity of the AT using ultrasound (Kubo et al., 2001; Lichtwark and Wilson, 2005; Peltonen et al., 2013; Suydam et al., 2015), suggesting that viscoelastic changes play a role in reducing the risk of AT injury (Wilson et al., 1991). Low hysteresis is advantageous for most tendons, indicating lower viscosity properties and excellent elastic properties that can effectively store and release elastic energy during activities, such as walking, running, and jumping (Peltonen et al., 2013). High hysteresis generates heat to increase temperature and may eventually lead to thermal injury and tendon degeneration during prolonged exercise (Wilson and Goodship, 1994). Given the importance of applied mechanical loading (stress and strain) on the adaptation of AT properties during running, exploring the effect of running exercise on the viscoelastic properties (e.g., hysteresis) of AT *in vivo* is crucial before exploring such effects in runners with different foot strike patterns.

Therefore, the objectives of this study are as follows: 1) to investigate the differences in male-specific AT morphological and viscoelastic properties between individuals with and without running exercise habits; 2) to examine the differences in male-specific AT morphological and viscoelastic properties between individuals with habitual foot strike patterns (RFS vs. FFS). We hypothesised that 1) runners had greater length, CSA, AT force, and lower hysteresis of AT than non-runners, and 2) FFS runners had greater length, CSA, force, and lower hysteresis of AT than RFS runners.

## 2 Materials and methods

### 2.1 Participants

The sample size was determined using G*Power software (3.0.1, Univ. Kiel, Kiel, Germany) through *a priori* power analysis based on preliminary data published by Intziegianni et al. (2016), which examined the differences in AT CSA between children, asymptomatic adults, and patients with Achilles tendinopathy (*f* = 0.63). The results indicated that a minimum sample size of 30 participants was required for a one-way analysis of variance (ANOVA) with a significance level *α* of 0.05 and a power *β* of 0.2. Therefore, this study recruited three groups consisting of 10 participants each: an RFS group, an FFS group, and a non-exercising control group (CG, Table 1). The runners in the RFS and FFS groups were required to have run a weekly mileage of more than 20 km in the past 4 weeks. The RFS group was accustomed to running with cushioned running shoes (featuring shock absorption and cushioning structures) in an RFS pattern, while participants in the FFS group were accustomed to running in an FFS pattern. The CG consisted of individuals who did not meet the minimum activity score in the International Physical Activity Questionnaire Short Form: i) engaging in vigorous physical activity for at least 20 min per day on at least 3 days or more in the past 7 days, ii) engaging in walking or moderate-intensity physical activity for at least 30 min per day on at least 5 days or more in the past 7 days or iii) engaging in a combination of walking, moderate-intensity, or high-intensity activities for at least 5 days or more in the past 7 days, with a minimum cumulative weekly total of 600 MET-minutes (Holler et al., 2019). All participants were males, right-leg dominant, and free from any lower limb injuries in the past year. They abstained from consuming caffeine and alcohol-containing food for 2 h prior to the tests, and they did not engage in vigorous or exhaustive exercise within 24 h preceding the tests. Participants were informed about the experimental procedures and objectives before testing and provided informed consent by signing a consent form. This study obtained ethical approval from the Ethics Committee of Shanghai University of Sport (approval no. 102772021RT085).

**TABLE 1:** Basic information of subjects in the habitual rearfoot striker (RFS) group, habitual forefoot striker (FFS) group, and non-exercise control group (CG, x̄ ± SD).

	CG (*n* = 10)	RFS group (*n* = 10)	FFS group (*n* = 10)
Age (years)	24.2 ± 2.9	33.1 ± 8.1	29.8 ± 9.5
Height (cm)	174.0 ± 3.4	174.1 ± 7.1	175.2 ± 5.4
Weight (kg)	71.9 ± 7.6	70.7 ± 9.7	71.2 ± 9.1

### 2.2 Procedure

Participants were instructed to change into experimental clothing consisting of a sports vest and shorts. They were asked to wear their own preferred running shoes and perform a 5-min warm-up exercise on the treadmill at a self-selected speed. At the beginning of the testing session, the initial length of the AT was measured using an ultrasound imaging device (uSmart 3300, Terason, United States). A detailed description of the model preparation was reported in our previous study (Zhang et al., 2021a). In brief, the following steps were performed: 1) participants were positioned in a prone position on the treatment table, the ankle joint was in a neutral position (90°), and the knee and hip joints were in an extended position (180°); 2) after the application of a coupling gel, a needle of size 25 was inserted between a 12L5A linear array probe (maximum frequency: 12 MHz; probe array length: 4.5 cm) and the skin surface to serve as a marker projection in the ultrasound image. The intersection point of the marker needle and the ultrasound probe on the skin was marked as the junction of the medial head of the gastrocnemius muscle and the AT (MTJ_GM_AT_); 3) the same procedure was repeated to mark the insertion point of the AT into the calcaneus; and 4) the distance between these two points was measured using a flexible ruler (Barfod et al., 2015). Subsequently, the ultrasound probe was positioned perpendicular to the skin surface to capture the AT CSA at a level consistent with the medial and lateral malleoli.

**FIGURE 1:**
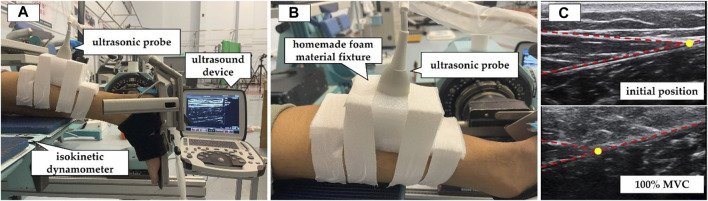
Measurement of mechanical properties of the Achilles tendon (AT) by synchronous ultrasound imaging with a maximum isometric contraction (MVC). **(A)** The participants were positioned prone on the CON-TREX isokinetic dynamometer. **(B)** The ultrasound probe was fixed at the junction between the AT and the medial head of the gastrocnemius muscle. **(C)** Ultrasound images of the AT in the resting position and during 100% MVC. The yellow circle indicated the location of the junction between the AT and the medial head of the gastrocnemius muscle.

Participants were instructed to lie prone on the isokinetic dynamometer (CON-TREX MJ, PHYSIOMED, Schnaittach, Freistaat Bayern, Germany) with their chest against the bed, hands naturally placed by their sides, hip and knee joints fully extended, and ankle joint in a neutral position (Figure 1A). They were asked to gradually increase their ankle joint dorsiflexion maximum voluntary isometric contraction (MVC) from relaxation to the maximum within 5 s and then gradually relax within 5 s (Kubo et al., 2001). During the testing process, the ultrasound probe was fixed at MTJ_GM_AT_ using an elastic bandage (Figure 1B). The real-time ultrasound video of the *in vivo* displacement changes in MTJ_GM_AT_ (Figure 1C) and the plantar flexion moment of the ankle joint during MVC were acquired simultaneously by connecting the ultrasound device and the isokinetic dynamometer via an external synchronisation box (BIOPAC Systems Inc., Goleta, CA, United States). The ultrasound video was measured at a sampling rate of 35 Hz, and the isokinetic dynamometer was measured at a sampling rate of 256 Hz. Before the test, the participants were given sufficient time to become familiar with the target tasks. Three sets of valid data were obtained, meeting the following criteria: 1) the participants exerted maximum effort to perform the MVC and 2) the ultrasound video capturing the displacement of MTJ_GM_AT_ during the MVC was clear. In the preliminary pre-experiment, the intraclass correlation coefficient (ICC) was used to evaluate the reliability of measurements by different experimenters and the same experimenter on different days. The results showed excellent reliability of the AT morphological properties as obtained and calculated (ICC = 0.895–0.996).

### 2.3 Data processing

ImageJ software (version 1.46r, NIH, United States) was used to evaluate the CSA images of the AT measured by tracing the surrounding echogenic boundary of the AT. Changes in the displacement of MTJ_GM_AT_ during MVC were semiautomatically tracked using the Tracker video modelling software (Tracker 4.95, InstallBuilder, United States). The position of MTJ_GM_AT_ was tracked automatically according to the in-house scripts of Tracker video modelling. Then, data were checked manually frame by frame, and the offset points were moved to the correct position. The aforementioned process was repeated three times to reduce errors, and the average values were obtained. After the manually checked data were acquired, linear interpolation was applied to adjust the sampling rate of the ultrasound image data sequences. The sampling rate was then changed to 256 Hz, which was consistent with the sampling frequency of the CON-TREX isokinetic dynamometer.

The plantar flexion moment (*M*_PF_) was obtained during MVC using the CON-TREX isokinetic dynamometer and normalised to the body weight (BW). Thus, F_AT_peak_ was calculated by deriving from the M_PF_ and moment arm (*L*_M_) of the AT as follows (Suydam et al., 2015):

$$F_{AT\_peak}=\frac{M_{PF}}{L_{M}},$$

where *F*_*AT_peak*_ is normalised by the BW and *L*_*M*_ is defined as the vertical distance from the centre point of the ankle joint to *AT*, which is set to a fixed value of 0.05 m (Rice and Patel, 2017).

The peak AT stress was calculated by dividing F_AT_peak_ by the CSA of the AT. The peak AT strain was defined as the length in MVC relative to the initial length and divided by the initial length. AT stiffness was calculated as the slope of the least-squares line of the ascending limb of the force–elongation curve between 50% and 100% of MVC force (Kubo et al., 2015). Hysteresis was calculated by subtracting the area under the descending limb of the force–elongation curve from the area under the ascending limb and dividing the difference by the area under the ascending limb (Peltonen et al., 2010).

### 2.4 Statistical analysis

All data were expressed as the mean ± standard deviation. The Shapiro–Wilk test was used to assess the normality of the data. One-way ANOVA was used to compare differences between the morphological (length and CSA) and mechanical characteristics of the AT (AT force, stress peak, strain peak, stiffness, and hysteresis) among the RFS group, FFS group, and CG. *Post hoc* pairwise multiple comparisons were conducted, the alpha Bonferroni was corrected, and the partial eta-squared (*η* ^2^) was applied as a measure of effect size. SPSS statistical software (IBM SPSS v22.0, IBM Corp., Armonk, NY, United States) was used for all statistical procedures with *p* < 0.05 as the statistical significance criterion.

## 3 Results

No significant differences in AT length and CSA were found among the RFS group, FFS group, and CG (*p* > 0.05, Table 2).

**TABLE 2:** Morphological properties of the Achilles tendon (AT) in the habitual rearfoot striker (RFS) group, habitual forefoot striker (FFS) group, and non-exercise control group (CG, x̄ ± SD).

Variable	CG (*n* = 10)	RFS group (*n* = 10)	FFS group (*n* = 10)	*p*-value	Partial *η* ^2^
AT length (cm)	20.3 ± 2.3	20.1 ± 3.3	20.6 ± 2.6	0.923	0.006
AT CSA (mm^2^)	61.4 ± 12.1	60.8 ± 6.0	64.1 ± 10.4	0.721	0.024

**FIGURE 2:**
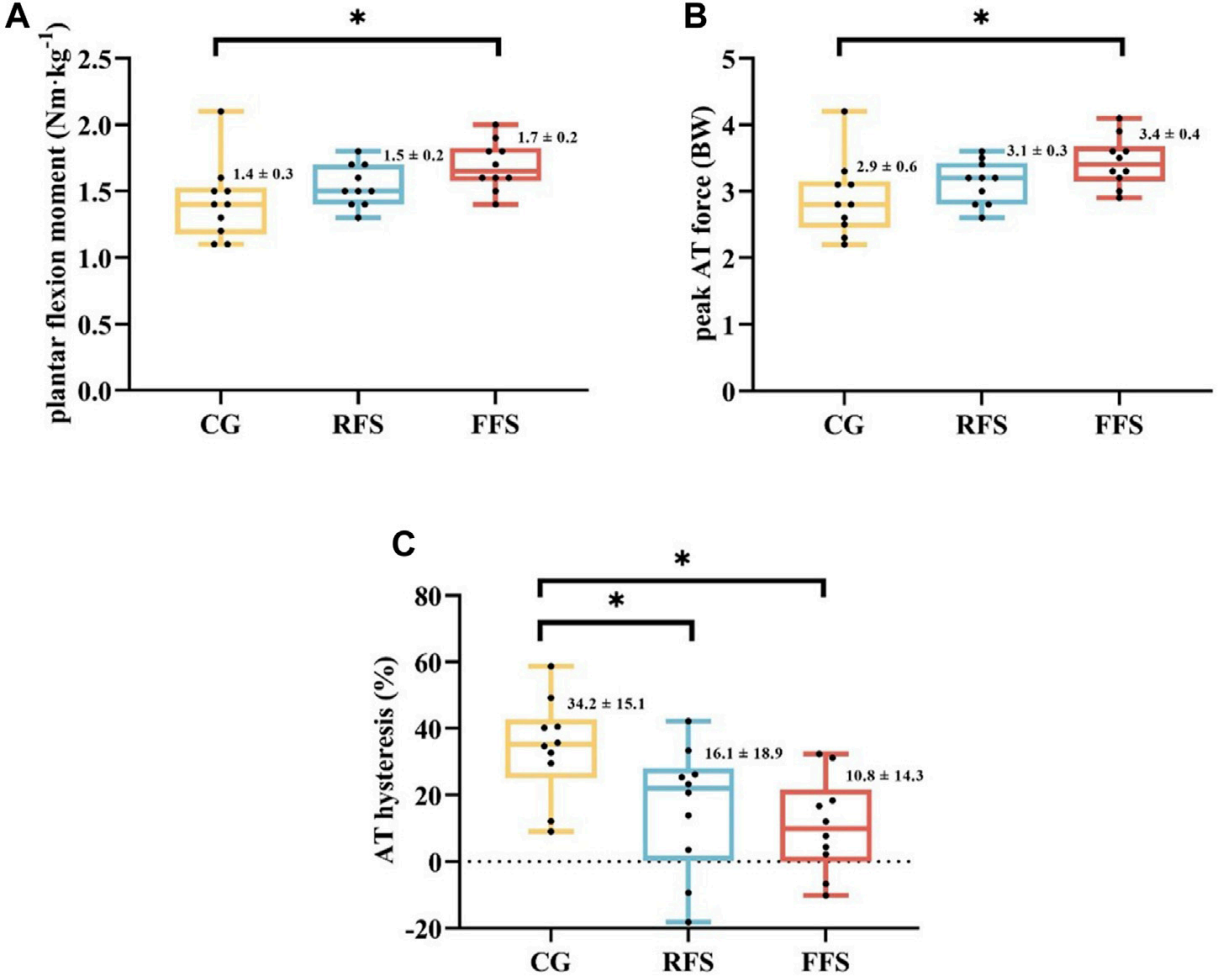
Differences in plantar flexor moment **(A)**, peak Achilles tendon (AT) force **(B)**, and AT hysteresis **(C)** at maximum isometric contraction among the habitual rearfoot striker (RFS) group, habitual forefoot striker (FFS) group, and non-exercise control group (CG). Scatter indicates individual data points for participants; * indicates *p* < 0.05.

Significant group differences were observed in the plantar flexion moment (*p* = 0.036 and *η* ^2^ = 0.219), F_AT_peak_ (*p* = 0.033 and *η* ^2^ = 0.223), and hysteresis of AT (*p* = 0.009 and *η* ^2^ = 0.297). *Post hoc* analysis revealed that the plantar flexion moment and F_AT_peak_ were significantly larger (21.4% and 17.2%, respectively) in the FFS group compared with the CG (*p* < 0.05, Figure 2). The AT hysteresis in the FFS group was significantly lower than that in the CG group by 68.4% (*p* < 0.05, Figure 2). However, no significant differences were observed between the FFS and RFS groups (*p* > 0.05, Table 3). Moreover, no significant differences in peak AT stress, peak AT strain, and stiffness were found among the three groups (*p* > 0.05, Table 3).

**TABLE 3:** Mechanical and viscoelastic properties of the Achilles tendon (AT) in the habitual rearfoot striker (RFS) group, habitual forefoot striker (FFS) group, and non-exercise control group (CG, x̄ ± SD).

Variable	CG (*n* = 10)	RFS group (*n* = 10)	FFS group (*n* = 10)	*p*-value	Partial *η* ^2^
Plantar flexor moment (Nm·kg^−1^)	1.4 ± 0.3	1.5 ± 0.2	1.7 ± 0.2^a^	0.036	0.219
Peak AT force (BW)	2.9 ± 0.6	3.1 ± 0.3	3.4 ± 0.4^a^	0.033	0.223
Peak AT stress (MPa)	34.2 ± 9.9	35.8 ± 6.6	38.1 ± 7.4	0.563	0.042
Changes in AT elongation (cm)	2.0 ± 0.2	2.1 ± 0.6	2.1 ± 0.4	0.669	0.029
Peak AT strain (%)	10.0 ± 1.6	10.6 ± 2.8	10.6 ± 2.8	0.782	0.018
AT stiffness (N·mm^−1^)	146.2 ± 66.6	134.0 ± 39.5	155.5 ± 41.3	0.640	0.032
AT hysteresis (%)	34.2 ± 15.1	16.1 ± 18.9	10.8 ± 14.3^a^	0.009	0.297

^a^ indicates significant differences between the FFS group and CG (*p* < 0.05) for *post hoc* tests.

## 4 Discussion

The purpose of this study was to investigate the morphological and viscoelasticity properties of the AT in RFS runners, FFS runners, and non-exercising individuals. Consistent with our hypothesis, the results revealed that the ankle plantar flexion moment and AT force during MVC were significantly greater and AT hysteresis was significantly lower in the FFS group than that in the CG. However, on the contrary to our study hypothesis, no significant differences were found between the FFS and RFS groups.

No significant differences in the AT length and CSA were found among the RFS group, FFS group, and CG. This result supported the study by Kubo et al. (2015), who observed no significant differences in AT morphology among habitual FFS runners, habitual RFS runners, and habitually midfoot strike runners. Compared with other tissues, tendons are generally considered metabolically inert and less prone to morphological changes (Merza et al., 2021). However, the study used magnetic resonance imaging (MRI) to investigate a significantly larger AT CSA (95 ± 3 mm^2^) in runners than in non-runners (73 ± 3 mm^2^; (Rosager et al., 2002). Similarly, Devaprakash et al. (2020) found that elite or sub-elite long-distance runners had a larger CSA and shorter AT length than healthy controls according to MRI. Furthermore, Histen et al. (2017) observed that habitual FFS male runners wearing minimalist shoes had a larger CSA of the AT compared with the habitual RFS male runners. The increased AT CSA has been indicated to be related to the changes in fibre diameter, fibre density, collagen protein, and proteoglycan content (Magnusson and Kjaer, 2019). However, repetitive tendon loading associated with running not only stimulates the increase in collagen and other matrix components but also affects their redistribution within a tendon. Some regions of a tendon may contain more matrix components, whereas others may contain fewer, which are susceptible to injury (Merza et al., 2021). Based on this theory, one possible reason for the inconsistency between our study and previous studies was the difference in measurement location. Our study and the study by Kubo et al. (2015) measured the AT CSA at the same level as the medial and lateral malleoli, whereas Rosager et al. (2002) measured the CSA at a point of 3 cm above the calcaneus. The CSA in the study by Devaprakash et al. (2020) was obtained by dividing the volume of the free AT by the tendon length. Additionally, a positive correlation between the AT CSA and body weight has been proposed (Magnusson et al., 2003; Kudron et al., 2020). Given that no significant difference in body weight was found among the participants in our study, there was no significant difference in the AT CSA among the groups, indicating that the AT CSA did not continuously increase within a certain range of body weight. The potential impact of body weight on different foot strike runners needs to be further explored in future research.

Stiffness was calculated from the linear portion of the force–elongation curve, and the selected region of the linear portion was currently inconsistent. In the present study, we elected 50%–100% regions, which were more appropriate for this study data (Kubo et al., 2007). Low AT stiffness has been shown to influence the speed of force transmission. Changes in energy expenditure are related to AT stiffness. The required AT stiffness during running was estimated to be approximately 250 N mm^−1^, higher than that during walking. Accordingly, we hypothesized that the AT of FFS runners was stiffer than that of RFS runners. However, despite male runners having lower levels of energy consumption (i.e., hysteresis) than male non-runners, no significant differences in AT stiffness were found among the three groups. Consistently, the previous study indicated no significant differences in AT stiffness among runners with different foot strike patterns [FFS vs. RFS vs. midfoot strike; (Kubo et al., 2015)]. Fletcher et al. (2013) investigated that energy expenditure measured using indirect calorimetry during running was only related to AT stiffness in females and not in male runners.

Mechanical loading produces adaptive changes in the AT, including increased morphological properties or mechanical properties (Devaprakash et al., 2020; Zhang et al., 2021b). Given the greater triceps muscle strength isrequired in habitual FFS runners during the stance phase, we hypothesized that the greater mechanical loading stimulated on the AT would be accompanied by an increase in the AT CSA and stiffness in the FFS group. However, our results indicated that the AT of FFS runners did not exhibit this adaptation, that is, no significant difference in AT morphological and viscoelastic properties between male runners with FFS and RFS were found. Only FFS male runners showed significantly higher AT force than male non-runners, and no differences were found between male runners with different foot strike patterns. Similar results were shown in the study by exploring AT loading in female runners (Kernozek et al., 2018). Greater AT force in FFS runners suggested more optimised athletic performance during the push-off phase of running, but asynchronous changes in mechanical and morphological properties may lead to a potential risk for Achilles tendinopathy.

Although the run itself did not affect the morphological properties of the AT, our study observed differences in the viscoelasticity properties of the AT among the three groups. In the cyclic force test (gradually increasing to 100% MVC over 5 s and gradually relaxing over 5 s), the loading and unloading curves produced a loop, which was defined as hysteresis. The area within this loop represented the heat loss due to internal damping, whereas the area under the unloading curve represented the energy restored during elastic recoil. The AT has good energy storage capacity and excellent elastic properties superior to its viscous properties (Peltonen et al., 2013), providing an energy-saving mechanism during human walking, running, and jumping. Furthermore, the results of this study showed that the FFS group had significantly lower AT hysteresis than the CG. The AT stored elastic energy during the early phase of support by elongation and released energy during the push-off phase of support to propel the body forward (Smith et al., 2022). A low hysteresis is advantageous for the AT because it indicates high elastic energy recovery and reduces energy loss during the movement while minimising heat-induced damage (Alexander, 2002). In the present study, the hysteresis values for the FFS group (10.8% ± 14.3%), RFS group (16.1% ± 18.9%), and CG (34.2% ± 15.1%) were consistent with the previous reports of hysteresis during running [2%–45% (Finni and Vanwanseele, 2023)] and during single-leg jumping [15%–39%; (Lichtwark and Wilson, 2005)]. The wide range of hysteresis variation may be attributed to several factors. First, it may be due to the anatomical structure of the tendon because the AT is a tendon shared by the three heads of the triceps surae. Each head can independently stretch the tendon, leading to regional strain variations. Despite efforts to reduce measurement errors by averaging multiple measurements, the uncertainty in measuring AT hysteresis is difficult to avoid when tendons are explored *in vivo* (Peltonen et al., 2013). Second, an individual’s ability to control motion during the unloading phase contributes to possible errors (Finni and Vanwanseele, 2023). Ultrasound sampling frequency is usually much lower than that of force measurements. Although this study corresponded to two datasets by interpolation, the effect of desynchronisation of force and ultrasound image on AT hysteresis is sensitive (Finni et al., 2013).

This study had several limitations. First, owing to the low sampling frequency of the ultrasound images, errors in the result are inevitable. Although this error may be resolved by averaging multiple trials from each participant, AT hysteresis is higher *in vivo* than *in vitro* and may be addressed only by validation studies, where the same AT is measured separately *in vivo* and *in vitro*. Second, this study only measured the viscoelastic properties of the AT at 100% MVC, and whether this property changes with foot strike pattern during running remains unclear. Finally, the assessment of AT morphological and viscoelastic properties is influenced by several factors, for example, synergistic muscle activity, AT and foot moment arm, preconditioning of the tendon, and the rate of force development. Therefore, the influences of these factors need to be further analysed. In addition, all participants were male in the current study, and whether female runners would show the same results remains unclear. In future studies, we suggest including female participants to better reflect the diversity of runners in the target population.

## 5 Conclusion

Habitual FFS exhibited significantly lower AT hysteresis than non-runners in men. This result suggested that long-term FFS patterns could enhance the male-specific AT’s ability to store and release elastic energy efficiently during running, resulting in a more effective stretch-shortening cycle. Furthermore, the peak AT force observed in the FFS group was greater than that in non-runners, suggesting that male FFS runners have a stronger Achilles tendon. However, foot strike patterns were not related to the morphological and viscoelastic properties of the AT in recreational male runners.

## Data Availability

The raw data supporting the conclusion of this article will be made available by the authors, without undue reservation.
